# Antireflux mucosectomy in the management of gastroesophageal reflux disease with large hiatal hernia

**DOI:** 10.1055/a-2638-6016

**Published:** 2025-07-15

**Authors:** Masayoshi Kure, Haruhiro Inoue, Kazuki Yamamoto, Yuto Shimamura, Yohei Nishikawa, Ippei Tanaka, Mayo Tanabe

**Affiliations:** 1 378609 Digestive Diseases Center, Showa Medical University Koto Toyosu Hospital, Koto, Japan

In recent years, minimally invasive treatments such as antireflux mucosectomy (ARMS) and antireflux mucosal ablation (ARMA) have shown efficacy in managing proton-pump-inhibitor–refractory gastroesophageal reflux disease (GERD) 1 2 3 . While surgical treatment is generally preferred for sliding hernias larger than 3 cm, the outcomes of ARMS in such cases remain unclear 4 5 . We report a case demonstrating the effectiveness of ARMS in treating GERD in a patient with a 3-cm sliding hernia.

A 68-year-old woman with a 15-year history of heartburn and chest pain was referred to our hospital after long-term vonoprazan use failed to alleviate her symptoms, despite ongoing antacid treatment. Upper endoscopy revealed grade C reflux esophagitis (Los Angeles classification) and a 3-cm sliding hiatal hernia ( Fig. 1 ). Although surgical fundoplication is typically recommended for large sliding hernias, this patient’s history of multiple laparotomies, chronic obstructive pulmonary disease, and pulmonary hypertension made her a high-risk candidate for surgery. As a result, ARMS was selected as a less invasive alternative.

**Fig. 1:**
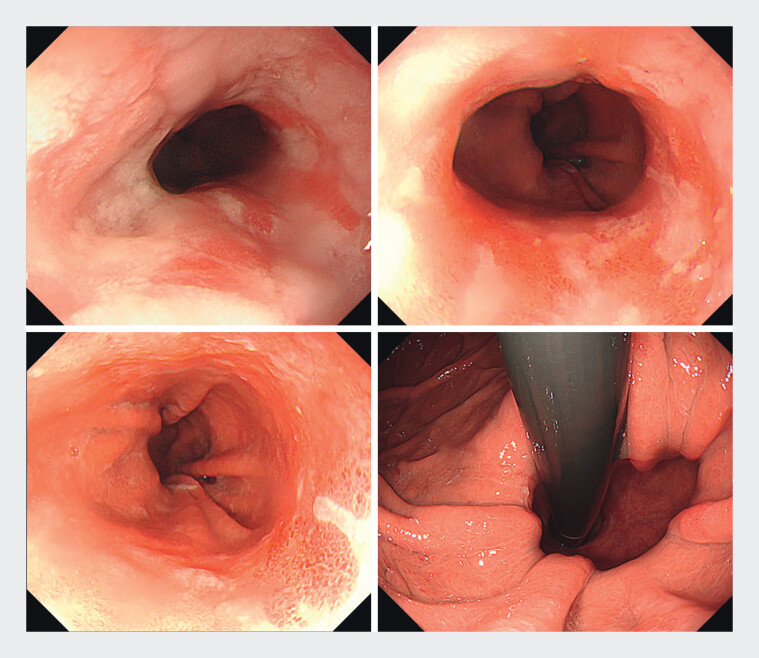
Grade C gastroesophageal reflux disease with a 3-cm sliding hiatal hernia in a 68-year-old woman with a 15-year history of heartburn and chest pain.

Cap-assisted endoscopic mucosal resection was performed from the cardia to the upper gastric body along the lesser curvature, excising four-fifths of the mucosal circumference, including within the hernia sac ( Fig. 2 , Video 1 ). The procedure was completed without adverse events, and the patient was discharged 9 days later. One month after the procedure, follow-up endoscopy showed a well-reconstructed gastroesophageal flap valve with no visible evidence of a hernia sac. Erosive GERD improved from grade C to grade M ( Fig. 3 ), and the patient’s GerdQ score significantly decreased from 14 before ARMS to 7 at 1 month after, and further to 2 at 2 months after. No postprocedural complications, such as bleeding or stenosis requiring dilation, were observed.

**Fig. 2:**
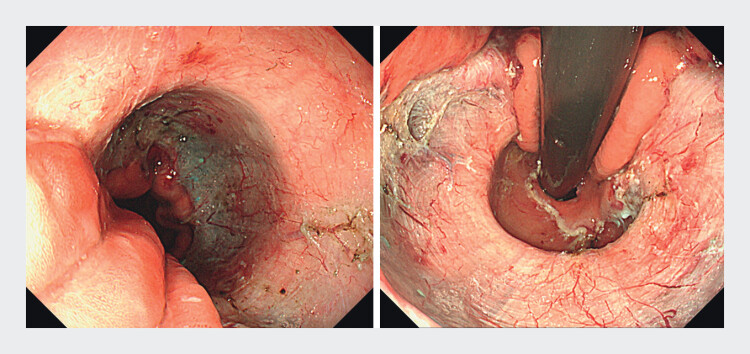
Cap-assisted endoscopic mucosal resection removed four-fifths of the mucosal circumference from the cardia to the upper body along the lesser curvature, including within the hernia sac.

Antireflux mucosectomy in the management of gastroesophageal reflux disease with large hiatal hernia.Video 1

**Fig. 3:**
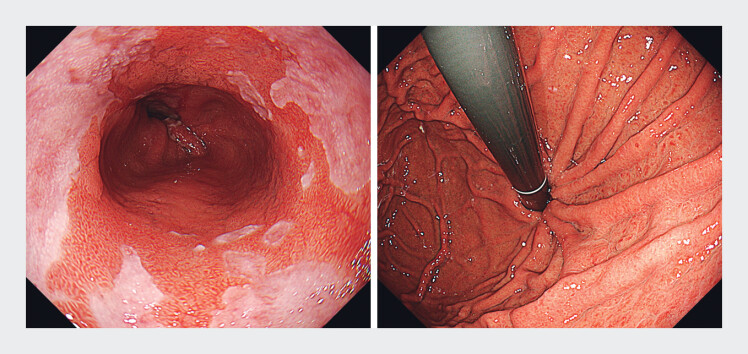
Follow-up endoscopy showed a well-reconstructed gastroesophageal flap valve with no hernia sac. Grade C gastroesophageal reflux disease improved to grade M.

These results suggest that ARMS may be an effective treatment option for GERD patients with a sliding hiatal hernia measuring 3 cm or larger. For patients with significant comorbidities for whom surgical treatment is deemed too invasive, ARMS offers a viable alternative.

Endoscopy_UCTN_Code_TTT_1AO_2AJ

## Notes

Endoscopy E-Videos https://eref.thieme.de/e-videos
*E-Videos* is an open access online section of the journal *Endoscopy* , reporting on interesting cases and new techniques in gastroenterological endoscopy. All papers include a high-quality video and are published with a Creative Commons CC-BY license. Endoscopy E-Videos qualify for HINARI discounts and waivers and eligibility is automatically checked during the submission process. We grant 100% waivers to articles whose corresponding authors are based in Group A countries and 50% waivers to those who are based in Group B countries as classified by Research4Life (see: https://www.research4life.org/access/eligibility/ ). This section has its own submission website at https://mc.manuscriptcentral.com/e-videos .
